# Intimate partner violence, depression, and sexual behaviour among gay, bisexual and other men who have sex with men in the PROUD trial

**DOI:** 10.1186/s12889-019-6757-6

**Published:** 2019-04-25

**Authors:** Ada R. Miltz, Fiona C. Lampe, Loraine J. Bacchus, Sheena McCormack, David Dunn, Ellen White, Alison Rodger, Andrew N. Phillips, Lorraine Sherr, Amanda Clarke, Alan McOwan, Ann Sullivan, Mitzy Gafos

**Affiliations:** 1 Centre for Clinical Research, Epidemiology, Modelling and Evaluation, Institute for Global Health, University College London, London, UK; 2 MRC Clinical Trials Unit, University College London, London, UK; 3 Department of Global Health and Development, London School of Hygiene and Tropical Medicine, London, UK; 4 Elton John Centre, Sussex House, Brighton, UK; 5 56 Dean Street, London, UK; 6 Chelsea & Westminster NHS Foundation Trust, London, UK

**Keywords:** Men who have sex with men (MSM), Intimate partner violence (IPV), Depression, Sexual risk behaviour, HIV, STI, Pre-exposure prophylaxis (PrEP)

## Abstract

**Background:**

Little is known about the prevalence and correlates of intimate partner violence (IPV) among gay, bisexual and other men who have sex with men (GBMSM) in the UK. The aim of this study was to investigate the prevalence of IPV, associations of socio-economic and psychosocial factors with IPV, and the association of IPV with depression and sexual behaviour, among GBMSM in the PROUD trial of pre-exposure prophylaxis (PrEP).

**Methods:**

PROUD enrolled 544 HIV-negative participants in England from 2012 to 2014; participants were randomised to immediate or deferred PrEP. This analysis included 436 GBMSM who had IPV data at month-12 and/or 24. Prevalence of IPV victimization and perpetration (lifetime, and in the past year) was assessed at these time-points. Generalized estimating equations were used to investigate associations with IPV, using pooled data from both time-points.

**Results:**

At month-12 (*N* = 410), 44.9% of men reported ever being a victim of IPV, 15.6% in the last year, and 19.5% reported ever perpetrating IPV, 7.8% in the last year. At month-24 (*N* = 333), the corresponding prevalence was 40.2 and 14.7% for lifetime and past year IPV victimization and 18.0 and 6.9% for lifetime and past year IPV perpetration. IPV prevalence did not differ by randomised arm. Men reporting internalized homophobia and sexualized drug use were more likely to report IPV. Lifetime and last year experience of IPV victimization and perpetration were strongly associated with depressive symptoms (PHQ-9 ≥ 10) (adjusted for socio-demographics: lifetime IPV victimization PR 2.57 [95% CI: 1.71, 3.86]; past year IPV victimization PR 2.93 [95% CI: 1.96, 4.40]; lifetime IPV perpetration PR 2.87 [95% CI: 1.91, 4.32]; past year IPV perpetration PR 3.47 [95% CI: 2.13, 5.64], *p* < 0.001 for all); IPV was not consistently associated with measures of condomless anal sex or high partner numbers.

**Conclusions:**

GBMSM at high-risk of HIV who are seeking/taking PrEP may experience a high burden of IPV, which may be linked to depression. Training on awareness of and enquiry for IPV among GBMSM in sexual health clinics is recommended.

**Trial registration:**

ClinicalTrials.gov identifier: NCT02065986. Registered 19 February 2014 (retrospectively registered).

**Electronic supplementary material:**

The online version of this article (10.1186/s12889-019-6757-6) contains supplementary material, which is available to authorized users.

## Background

Intimate partner violence (IPV) is defined as physical, sexual, or psychological harm by a current or former partner or spouse [1]. IPV may be experienced as a victim or perpetrator, or as both, often referred to as bidirectional/reciprocal IPV [2]. There is a growing body of research on the prevalence of IPV victimization among gay, bisexual and other men who have sex with men (GBMSM) in the U.S. [3–5], although data on IPV perpetration are limited.

Few studies have investigated IPV among GBMSM in the UK [6–8]. In a study of GBMSM attending a genitourinary medicine (GUM) clinic in London in 2010–11 (*N* = 519) [7], the prevalence of lifetime IPV victimization was 34% and lifetime IPV perpetration was 16%, which appears high in relation to general UK population estimates [9, 10].

Evidence from a recent meta-analysis of mainly U.S. studies suggests that experiences of IPV are associated with depression, drug use, sexual risk behaviour, and HIV seropositivity among GBMSM [3]. It has been suggested in syndemic theory that it is the synergistic interaction of two or more co-occurring psychosocial factors, such as childhood sexual abuse, IPV, depression, and drug use that may compound the risk of HIV/STIs [11, 12].

The arena of HIV prevention has changed dramatically with the introduction of pre-exposure prophylaxis (PrEP) medication to prevent HIV infection [13–15]. Although PrEP is not currently freely available on the UK National Health Service, an increasing number of GBMSM are using PrEP in the UK. The PROUD clinical trial evaluated the efficacy of PrEP against HIV acquisition among GBMSM in England [15, 16]. One concern of the trial was that PrEP use could potentially negatively impact upon vulnerability towards pressure from a partner to have sex without a condom. Therefore, the PROUD trial included inquiry on sexual, psychological, and physical IPV on the annual questionnaires, and whether participation in the trial had influenced experiences of IPV.

This analysis uses data from the 12- and 24-month follow-up of the PROUD trial. The aim is to investigate among HIV-negative GBMSM at high-risk for acquiring HIV infection, and in the context of PrEP use: (i) prevalence of IPV victimization and perpetration, and the impact of participating in a PrEP trial on experiences of IPV; (ii) associations of socio-economic status and psychosocial factors with IPV; (iii) association of IPV with depressive symptoms; (iv) relationships of IPV and depression with sexual behaviours.

## Methods

The PROUD trial was a multi-centre, pragmatic open label randomised clinical trial evaluating the benefit of PrEP as part of a package of HIV risk reduction interventions for HIV-negative GBMSM and trans women. Only three trans women enrolled in PROUD, and therefore data cannot be presented separately for trans women. The study was reviewed and approved by London Bridge Research Ethics Committee. Participants were enrolled at 13 GUM clinics in England between November 2012 and April 2014. Volunteers were eligible if they met the following criteria: male at birth, aged 18 years or over, tested HIV-negative on the day of enrolment or in the past four weeks, and reported condomless anal sex (CAS) with a man in the past three months and expected to have CAS again in the next three months [15]. Volunteers were randomized 1:1 to an immediate start of daily oral PrEP or a deferred start after 12 months of follow-up. However, during follow-up, an unexpectedly high incidence of HIV was observed in the deferral arm (9.0 per 100 person-years, 90% CI: 6.1, 12.8), which led to the decision by the trial steering committee in October 2014 to offer all participants PrEP. Participants had the opportunity to remain in follow-up for at least two years. Participants were asked to self-complete a paper questionnaire at baseline and an extended questionnaire, excluding socio-demographic data, on an approximately annual basis thereafter.

### IPV

**Fig. 1:**
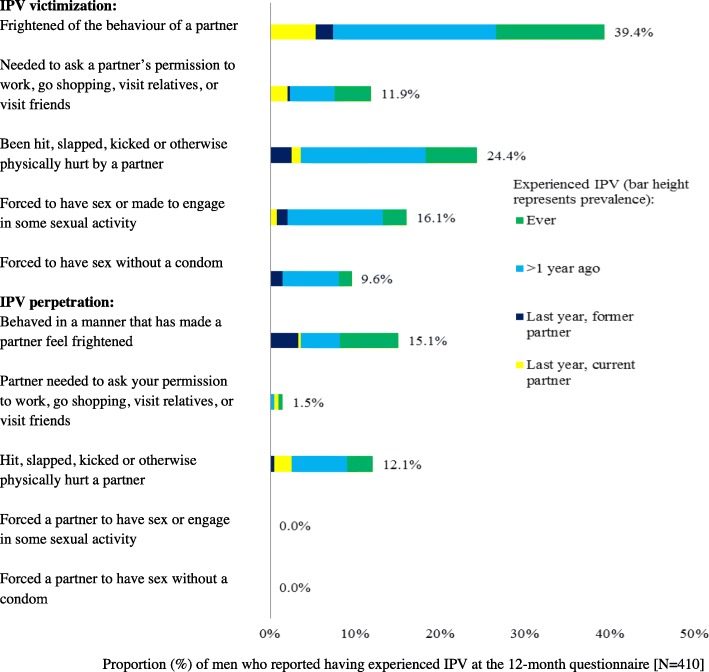
Prevalence of IPV victimization and perpetration at month-12 (*N* = 410)

**Fig. 2:**
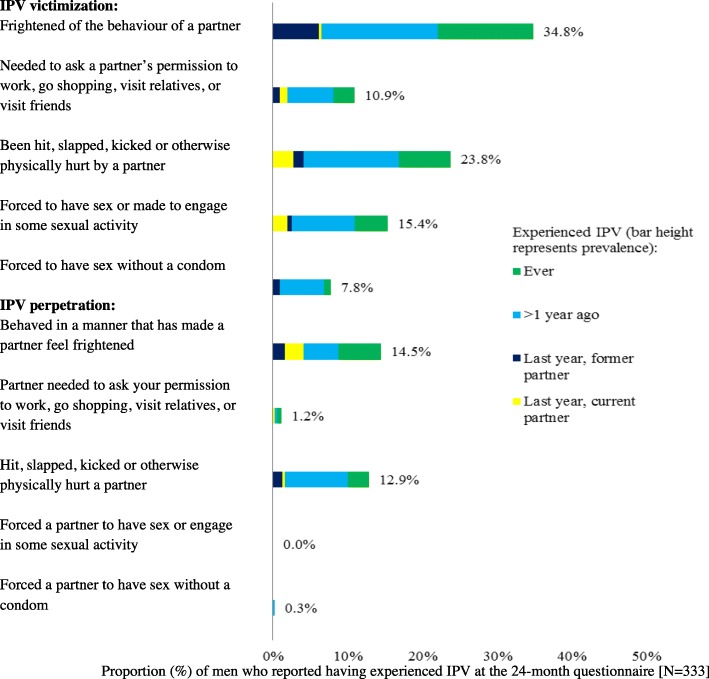
Prevalence of IPV victimization and perpetration at month-24 (*N* = 333)

Questions on IPV were not included in the baseline questionnaire but were included in the annual questionnaires, given concern over the possible impact of PrEP use on vulnerability towards pressure from partners to have sex without a condom. The IPV questions were based on the ‘Health and Relationships survey’ devised as part of a previous study in London [7]. Ten questions about psychological, physical, and sexual IPV were incorporated into the PROUD 12 and 24-month questionnaires. Five asked about victimization and five about perpetration; in each case response options differentiated experiences of lifetime IPV or IPV in the last year, and IPV with a current or former partner in the last year (Figs. 1 and 2 and question 19 in Additional file 1). A positive response to any one of the five respective questions was considered to indicate lifetime IPV victimization or lifetime IPV perpetration. Reporting any IPV victimization but no IPV perpetration was considered to indicate unidirectional IPV victimization. Likewise, the reverse was considered to indicate unidirectional IPV perpetration. A missing response was considered to indicate no IPV. In order to evaluate the impact of participating in the PROUD trial on IPV (including sexual violence), participants were asked the following question after enquiry about IPV; ‘If you answered yes in the last year to any question above, do you think joining PROUD has influenced these behaviours?’

### Clinically significant depressive symptoms

The Patient Health Questionnaire (PHQ-9) was used to measure the prevalence of depressive symptoms at month-12 and 24 (see question 16 in Additional file 1) [17]. In this analysis, a cut-off point of 10 or greater (out of a possible 27) for the total PHQ-9 score across the nine questions was used to indicate depressive symptoms. The PHQ-9 has been found to have good validity in a number of studies [17–28].

### Sexual behaviour measures

Seven measures of sexual behaviour in the past three months were derived at month-12 and 24; five measures of CAS and two measures of partner numbers. (i) CAS with at least two receptive or at least two insertive partners, (ii) CAS with at least five receptive or at least five insertive partners, (iii) CAS with an HIV-positive partner not known to be on antiretroviral treatment, (iv) receptive CAS with an HIV-positive partner not known to be on antiretroviral treatment, (v) most recent CAS with an unknown/HIV-positive partner not known to be on antiretroviral treatment, (vi) ten or more new anal sex partners, and (vii) receptive anal sex with ten or more partners. In measures (i) and (ii), numbers of insertive and receptive partners reported were considered separately as participants may have referred to the same partner for both insertive and receptive CAS. For instance, participants were not classified as positive for CAS with ≥2 partners if they reported one receptive and one insertive CAS partner (*n* = 37 at month-12 and *n* = 29 at month-24). In measures (vi) and (vii), a cut-off of ten or more partners was chosen given the very high prevalence of reporting five or more partners: 44.6% for new anal sex partners and 51.5% for receptive anal sex partners. A missing response was considered to indicate that the sexual behaviour did not occur.

### Use of drugs in a sexual context

At month-12 and 24, participants were asked whether, in the past three months, they had engaged in sex after recreational drug use, referred to as sexualized drug use. A missing response was considered to indicate no sexualized drug use. Sexualized use of drugs may be a proxy measure for chemsex, which is the use of specific psychoactive substances (usually one or more of mephedrone, gamma-hydroxybutrate/gamma-butyrolactone [GHB/GBL] and methamphetamine) during/immediately before sex to stimulate sexual arousal [29].

### Age at first anal sex

Data on age at anal sex debut with a male were collected at month-12 and 24. The UK Sexual Offences Act 2003 stipulates the legal age of sexual consent to be 16 and that children under age 13 have no legal capacity to consent to any form of sexual activity since they cannot fully comprehend nor are they developmentally prepared for it [30]. Two measures of age at anal sex debut were investigated, including cut-offs of age < 13 and age ≤ 15. Reports of sexual intercourse before age 13 may include experiences of childhood sexual abuse (CSA), however, since information was not collected on the age of the sexual partner and whether they felt forced, it is possible that some participants may not have experienced forced/coerced sex. Furthermore, experiences of CSA may occur at ages older than 13 years. Therefore, although it was of interest in this analysis to investigate a very young age at sexual debut as this measure may include many cases of CSA, it may not reflect or pick up all sexual abuse experienced.

### Internalized homophobia

The concept of internalized homophobia is described in Meyer’s minority stress theory as a consequence of perpetual negative feedback and shaming in the form of homophobic stereotyping and prejudice [31]. Eight questions about attitudes towards gay sexuality were asked at month-12 and 24 (see question 22 in Additional file 1) based on the 26-item Internalized Homophobia Scale (IHS) [32]. Reporting one or more negative attitudes towards gay sexuality (agree/disagree depending on the phrasing of the question) was considered to be indicative of internalized homophobia. A missing response was considered to indicate no internalized homophobia.

### Statistical analysis

The analysis is based on PROUD participants who completed either the 12- or 24-month questionnaire. Results are presented in the tables for lifetime and last year experiences of IPV victimization (yes or no/missing) and lifetime and last year experiences of IPV perpetration (yes or no/missing). Experiences of IPV victimization and perpetration were also categorized into a single variable as follows: neither victimization nor perpetration, unidirectional IPV victimization; unidirectional IPV perpetration; both victimization and perpetration. Prevalence and 95% confidence interval (CI) of the IPV measures were assessed. The main analysis of factors associated with IPV was based on pooled data from the 12- and 24-month time-points (as IPV and psychosocial factors were collected at both time-points) and used generalized estimating equations (GEE). Individuals who contributed data at both time-points were included in the GEE model twice; the use of robust standard errors account for non-independence of responses. GEEs were fitted using Poisson models with a log link in order to produce prevalence ratios (PRs) and compound symmetry for the correlational structure [33, 34]. For socio-demographic factors that were only collected at baseline, baseline values were used throughout, with the exception of age, for which age at the specific questionnaire completion was used. Associations of socio-demographic and psychosocial factors with IPV measures are presented unadjusted and adjusted for key socio-demographic factors assumed not to be on the causal pathway. These were: age [< 25, 25–29, 30–39, 40+ years], country of birth [UK born or non-UK born/missing], university education [yes or no/missing]), sexual identity [gay or bisexual/straight], and study region [London or outside London]. Associations of IPV measures with depressive symptoms, and with sexual behaviours were assessed unadjusted, and adjusted for the above socio-demographic factors, using Poisson GEE models. Of note, as indicated in the results, the data were not analyzed longitudinally given that the number of incident reports of IPV (i.e. no report of IPV at month-12 but IPV reported at month-24) was small.

Given that previous studies have linked depression to IPV and to sexual risk behaviour, further analyses were performed in order to examine the role of depressive symptoms on the relationship between IPV and sexual behaviour, and findings are briefly described in the text. These analyses investigated associations of: (i) depression with sexual behaviours in unadjusted and socio-demographic adjusted GEE models, (ii) IPV measures with sexual behaviours adjusted additionally for depression in GEE models, and (iii) lifetime IPV victimization with sexual behaviours among men with depression and among men without depression, via use of interaction terms in unadjusted GEE models.

For each measure and at each time-point, the proportion of missing responses was < 5%. For sexual behaviours, psychosocial measures (sexualized drug use, internalized homophobia, and IPV), and socio-economic factors (university education and employment), missing responses were considered to indicate that the event did not occur, as there appeared to be a common response pattern in which only those experiences that had occurred were ticked on the questionnaire. A sensitivity analysis was undertaken excluding missing values when defining each variable. All analyses were performed in STATA statistical software (version 13) [35] and reported according to the STROBE guidelines [36].

## Results

In total, 540 of the 544 participants enrolled in PROUD completed a baseline questionnaire, 410 completed a 12-month questionnaire, and 333 completed a 24-month questionnaire. The current analysis is based on 436 men who completed either a 12- or 24-month questionnaire (743 questionnaire responses in total). The vast majority of participants who completed the 12-month questionnaire reported being gay (95.6%) and white ethnicity (82.2%). The median age was 37 years (Interquartile range [IQR]: 31–44 years). Forty percent of men were born outside the UK and the majority reported university degree level education (62.4%), being employed (81.0%), and attending a study clinic in London (70.5%) (Table 1). Socio-demographic characteristics of the participants who completed the 24-month questionnaire were very similar.

**Table 1:** Adjusted associations with lifetime IPV victimization and IPV victimization in the last year, using pooled 12/24-month data

*N* = 743 observations (using pooled 12/24 data in GEE models; *N* = 436 men)		Lifetime IPV victimization		IPV victimization in the last year	
		% (n/N)	Adjusted^a^ PR [95% CI] *Overall p value* ^c^	% (n/N)	Adjusted^a^ PR [95% CI] *Overall p value* ^c^
Study time-point	Month-12	44.9% (184/410)	1	15.6% (64/410)	1
	Month-24	40.2% (134/333)	0.92 [0.76, 1.10]	14.7% (49/333)	0.98 [0.69, 1.40]
			*0.36*		*0.93*
Randomized to study trial arm	Immediate	42.0% (167/398)	1	15.8% (63/398)	1
	Deferred	43.8% (151/345)	1.05 [0.82, 1.35]	14.5% (50/345)	0.89 [0.60, 1.32]
			*0.70*		*0.57*
London study clinic site	Yes	40.6% (210/517)	1	14.3% (74/517)	1
	No	47.8% (108/226)	1.18 [0.89, 1.56]	17.3% (39/226)	1.31 [0.85, 2.02]
			*0.24*		*0.23*
Age	< 25	55.3% (21/38)	1.27 [0.75, 2.15]	23.7% (9/38)	1.61 [0.73, 3.59]
	25–29	48.4% (44/91)	1.19 [0.80, 1.77]	19.8% (18/91)	1.32 [0.71, 2.44]
	30–34	39.4% (63/160)	0.94 [0.65, 1.36]	13.1% (21/160)	0.83 [0.45, 1.51]
	35–39	47.2% (68/144)	1.10 [0.76, 1.59]	16.0% (23/144)	1.02 [0.57, 1.84]
	40–44	35.0% (44/125)	0.86 [0.58, 1.28]	11.2% (14/125)	0.74 [0.38, 1.44]
	45+	42.2% (78/185)	1	15.1% (28/185)	1
			*0.61*		*0.44*
			*0.31* ^b^		*0.27* ^b^
Born in the UK and ethnicity (BAME = Black, Asian, and minority ethnic)	Yes, white	45.0% (183/407)	1	14.3% (58/407)	1
	Yes, BAME	51.3% (20/39)	1.18 [0.70, 1.99]	20.5% (8/39)	1.40 [0.63, 3.10]
	No, white	39.1% (79/202)	0.90 [0.66, 1.23]	12.9% (26/202)	0.97 [0.58, 1.62]
	No, BAME	37.6% (35/93)	0.84 [0.54, 1.31]	22.6% (21/93)	1.83 [1.04, 3.20]
			*0.69*		*0.15*
Self-reported sexual identity	Gay	43.1% (303/703)	1	15.2% (107/703)	1
	Bisexual/ straight^d^	29.4% (10/34)	0.62 [0.29, 1.30]	14.7% (5/34)	0.84 [0.32, 2.22]
			*0.21*		*0.73*
University Education	Yes	42.0% (193/460)	1	15.7% (72/460)	1
	No/missing	44.2% (125/283)	1.01 [0.77, 1.32]	14.5% (41/283)	0.84 [0.55, 1.29]
			*0.96*		*0.43*
Employed	Yes	41.3% (251/608)	1	14.5% (88/608)	1
	No/missing	49.6% (67/135)	1.17 [0.85, 1.62]	18.5% (25/135)	1.17 [0.70, 1.94]
			*0.33*		*0.55*
Had sex after using recreational drugs (past three months)	No/missing	38.1% (137/360)	1	10.8% (39/360)	1
	Yes	47.3% (181/383)	1.36 [1.08, 1.71]	19.3% (74/383)	1.92 [1.28, 2.90]
			***0.010***		***0.002***
Group sex (past three months)	No/missing	42.1% (150/356)	1	11.0% (39/356)	1
	Yes	43.4% (168/387)	1.10 [0.88, 1.38]	19.1% (74/387)	1.87 [1.25, 2.79]
			*0.38*		***0.002***
Age < 13 years at anal sex debut	No	43.7% (295/675)	1	15.3% (103/675)	1
	Yes	53.7% (22/41)	1.15 [0.72, 1.84]	22.0% (9/41)	1.49 [0.74, 3.03]
			*0.57*		*0.27*
Age ≤ 15 years at anal sex debut	No	42.3% (236/558)	1	14.7% (82/558)	1
	Yes	51.3% (81/158)	1.18 [0.89, 1.56]	19.0% (30/158)	1.33 [0.85, 2.08]
			*0.26*		*0.21*
Negative attitudes towards gay sexuality	No/missing	38.3% (166/434)	1	10.8% (47/434)	1
	Yes	49.2% (152/309)	1.31 [1.05, 1.64]	21.4% (66/309)	2.00 [1.36, 2.94]
			***0.016***		***< 0.001***
‘Out’ to all/almost all friends, work mates, and close family	Yes	44.6% (166/372)	1	14.0% (52/372)	1
	No	42.3% (150/355)	1.08 [0.86, 1.36]	17.2% (61/355)	1.25 [0.85, 1.85]
			*0.52*		*0.25*
Lifetime IPV perpetration	No/missing	32.0% (193/603)	1	8.8% (53/603)	1
	Yes	89.3% (125/140)	2.69 [2.11, 3.42]	42.9% (60/140)	4.72 [3.22, 6.93]
			***< 0.001***		***< 0.001***

^a^Age (< 25, 25–29, 30–39, 40+), born in the UK, sexual identity (gay or bisexual/straight), university education, and London study clinic site

^b^Test for trend

^c^p value by Wald test using GEEs. *p* values< 0.1 are indicated in bold

^d^Five men identified as straight (1.2%)

### Prevalence of IPV

In Figs. 1 and 2, the prevalence of psychological, physical, and sexual IPV victimization and perpetration at month-12 and 24 are presented. Prevalence is described below for the 12-month data; results were similar at month-24. Overall, 44.9% (184/410; 95% CI: 40.1, 49.7%) of participants reported IPV victimization in their lifetime and 15.6% (64/410; 95% CI: 12.4, 19.5%) in the last year. The most common form of victimization was being frightened of a partner’s behaviour, reported by 39.4% (*n* = 154) of men in their lifetime. Having experienced physical violence from a partner was also common, being reported by 24.4% (*n* = 96) of men. Sixteen percent (*n* = 63) of participants reported having been forced to have sex (‘*Have you ever been forced to have sex or made to engage in some sexual activity when you did not want to’*) and 10% (*n* = 38) reported having been forced to have sex without a condom (*‘Have you ever been forced to have sex without a condom when you did not want to’*) in their lifetime. In total, 19.5% (80/410; 95% CI: 15.9, 23.7%) of participants reported IPV perpetration in their lifetime and 7.8% (32/410; 95% CI: 5.6, 10.8%) in the last year. Having behaved in a manner that frightened a partner and having been physically violent were the most common forms of IPV perpetration, at 15.1% (*n* = 59) and 12.1% (*n* = 48) respectively. With the exception of one man at the 24-month follow-up, all individuals responded with ‘never’ to the two sexual IPV perpetration questions (see Figs. 1 and 2). Overall, at month-12, 16.8% of men (69/410; 95% CI: 13.5, 20.8%) reported both lifetime IPV victimization and lifetime IPV perpetration. Unidirectional IPV victimization was reported by 28.1% of men (155/410; 95% CI: 23.9, 32.6%), and unidirectional IPV perpetration by 2.7% (11/410; 95% CI: 1.5, 4.8%). Due to small numbers, unidirectional IPV perpetration was not investigated in further analyses.

In terms of changes in individual IPV status between time-points among the 307 men who completed both the 12- and 24-month questionnaire; of 134 men who reported lifetime IPV victimization at month-12, 85 (63.4%) continued to report this measure of IPV at month-24. Of 58 men who reported lifetime IPV perpetration at month-12, 35 (60.3%) continued to report this measure of IPV at month-24. Of the 173 men who did not report lifetime IPV victimization at month-12, 39 (22.5%) reported it at month-24. Of the 249 men who did not report lifetime IPV perpetration at month-12, 19 (7.6%) reported it at month-24. Of note, of 47 men who reported IPV victimization in the last year at month-12, 14 (29.8%) reported it again at month-24. Of the 24 men who reported IPV perpetration in the last year at month-12, 6 (25.0%) reported it again at month-24.

At month-12, of the 70 participants who reported IPV victimization or perpetration within the last year, 72.9% (51/70) reported that joining PROUD had not influenced IPV behaviours, 21.4% (15/70) reported it had influenced them in a positive way, and no one reported it had influenced them in a negative way (5.7% had a missing response). At month-24, the equivalent data showed that 65.5% (36/55) reported that joining PROUD had not influenced IPV behaviours, 18.2% (10/55) reported it had influenced them in a positive way, and 3.6% (2/55) in a negative way (12.7% had a missing response). The two men who reported a negative influence reported IPV at month-24 but not month-12. Both reported being frightened of the behaviour of a current/former partner in the last year, and one of them reported being forced to have sex in the last year with a current partner.

### Association of socio-demographic and psychosocial factors with IPV

Table 1 shows adjusted associations with lifetime IPV victimization and IPV victimization in the last year. Associations are adjusted for age, born in the UK, sexual identity, university education, and London study clinic site. Unadjusted PRs were very similar to the adjusted PRs presented. Lifetime and past year measures of IPV victimization were strongly associated with sexualized drug use, a marker of internalized homophobia, and lifetime IPV perpetration, and for past year experiences of IPV only, group sex. Of note, of the 224 men who reported sexualized drug use at month-12, 67.9% had reported the use of drugs most commonly associated with chemsex (mephedrone, GHB/GBL, and/or methamphetamine) in the past three months at baseline. Measures of IPV victimization were not associated with study time-point, trial arm, clinic site, age, country of birth/ethnicity, sexual identity, university education, employment, age < 13 or age ≤ 15 years at anal sex debut, or being out to all friends/work colleagues/close family.

Adjusted associations with lifetime IPV perpetration and IPV perpetration in the last year are shown in Table 2. Lifetime and past year measures of IPV perpetration were strongly associated with younger age and sexualized drug use. The prevalence of lifetime IPV perpetration was more than eight times higher in men who reported IPV victimization, and the prevalence of past year IPV perpetration was almost 14 times higher in men who reported IPV victimization, compared to men who did not. There was some evidence that internalized homophobia was associated with lifetime IPV perpetration, and a strong association was found with past year experiences of IPV perpetration. There was some evidence that group sex was associated with lifetime IPV perpetration, but not IPV perpetration in the last year.

**Table 2:** Adjusted associations with lifetime IPV perpetration and IPV perpetration in the last year, using pooled 12/24-month data

*N* = 743 observations (using pooled 12/24 data in GEE models; *N* = 436 men)		Lifetime IPV perpetration		IPV perpetration in the last year	
		% (n/N)	Adjusted^a^ PR [95% CI] *Overall p value*^c^	% (n/N)	Adjusted^a^ PR [95% CI] *Overall p value*^c^
Study time-point	Month-12	19.5% (80/410)	1	7.8% (32/410)	1
	Month-24	18.0% (60/333)	0.98 [0.76, 1.25]	6.9% (23/333)	0.99 [0.58, 1.68]
			*0.86*		*0.98*
Randomized to study trial arm	Immediate	19.9% (79/398)	1	7.8% (31/398)	1
	Deferred	17.7% (61/345)	0.89 [0.60, 1.32]	7.0% (24/345)	0.81 [0.46, 1.41]
			*0.56*		*0.45*
London study clinic site	Yes	18.4% (95/517)	1	7.9% (41/517)	1
	No	19.9% (45/226)	1.01 [0.65, 1.57]	6.2% (14/226)	0.71 [0.37, 1.35]
			*0.99*		*0.30*
Age	< 25	34.2% (13/38)	2.12 [1.04, 4.30]	18.4% (7/38)	5.53 [1.80, 17.0]
	25–29	31.9% (29/91)	1.79 [1.00, 3.20]	19.8% (18/91)	5.89 [2.30, 15.10]
	30–34	15.0% (24/160)	0.92 [0.50, 1.70]	5.6% (9/160)	1.73 [0.60, 4.97]
	35–39	18.8% (27/144)	1.25 [0.69, 2.24]	7.6% (11/144)	2.26 [0.81, 6.28]
	40–44	16.0% (20/125)	0.98 [0.53, 1.82]	3.2% (4/125)	0.98 [0.27, 3.57]
	45+	14.6% (27/185)	1	3.2% (6/185)	1
			*0.09*		***< 0.001***
			***0.034*** ^**b**^		***< 0.001*** ^**b**^
Born in the UK and white ethnicity (BAME = Black, Asian, and minority ethnic)	Yes, white	19.2% (78/407)	1	7.1% (29/407)	1
	Yes, BAME	25.6% (10/39)	1.06 [0.48, 2.35]	12.8% (5/39)	1.10 [0.40, 3.05]
	No, white	17.8% (36/202)	1.05 [0.65, 1.70]	6.4% (13/202)	0.97 [0.48, 1.95]
	No, BAME	17.2% (16/93)	0.92 [0.47, 1.78]	8.6% (8/93)	1.13 [0.49, 2.61]
			*0.98*		*0.99*
Self-reported sexual identity	Gay	18.9% (133/703)	1	7.4% (52/703)	1
	Bisexual/straight^d^	17.7% (6/34)	0.79 [0.30, 2.08]	8.8% (3/34)	0.91 [0.27, 3.04]
			*0.63*		*0.88*
University Education	Yes	23.0% (65/283)	1	9.2% (26/283)	1
	No/missing	16.3% (75/460)	1.31 [0.87, 1.96]	6.3% (29/460)	1.28 [0.73, 2.25]
			*0.19*		*0.39*
Employed	Yes	19.4% (118/608)	1	7.7% (47/608)	1
	No/missing	16.3% (22/135)	0.68 [0.39, 1.20]	5.9% (8/135)	0.60 [2.67, 1.34]
			*0.18*		*0.21*
Had sex after using recreational drugs (past three months)	No/missing	12.5% (45/360)	1	4.2% (15/360)	1
	Yes	24.8% (95/383)	1.75 [1.23, 2.50]	10.4% (40/383)	2.16 [1.17, 3.96]
			***0.002***		***0.013***
Group sex (past three months)	No/missing	15.7% (56/356)	1	6.5% (23/356)	1
	Yes	21.7% (84/387)	1.38 [1.00, 1.91]	8.3% (32/387)	1.42 [0.82, 2.46]
			***0.050***		*0.21*
Age < 13 years at anal sex debut	No	19.6% (132/675)	1	7.6% (51/675)	1
	Yes	17.0% (7/41)	1.01 [0.48, 2.13]	7.3% (3/41)	1.06 [0.33, 3.44]
			*0.98*		*0.92*
Age ≤ 15 years at anal sex debut	No	18.1% (101/558)	1	7.2% (40/558)	1
	Yes	24.1% (38/158)	1.32 [0.88, 1.99]	8.9% (14/158)	0.93 [0.48, 1.77]
			*0.18*		*0.82*
Negative views about gay sexuality	No/missing	16.8% (73/434)	1	5.1% (22/434)	1
	Yes	21.7% (67/309)	1.33 [0.97, 1.83]	10.7% (33/309)	2.10 [1.21, 3.65]
			***0.075***		***0.008***
‘Out’ to all/almost all friends, work mates, and close family	Yes	22.6% (84/372)	1	8.1% (30/372)	1
	No	15.8% (56/355)	0.79 [0.56, 1.12]	7.0% (25/355)	0.93 [0.53, 1.62]
			*0.19*		*0.79*
Any IPV victimization	No/missing	3.5% (15/425)	1	1.2% (5/425)	1
	Yes	39.3% (125/318)	8.47 [5.09, 14.09]	15.7% (50/318)	13.54 [5.33, 34.38]
			***< 0.001***		***< 0.001***

^a^Age (included as four categories: < 25, 25–29, 30–39, 40+), born in the UK, sexual identity (gay or bisexual/straight), university education, and London clinic site

^b^Test for trend

^c^p value by Wald test using GEEs. *p* values< 0.1 are indicated in bold

^d^Five men identified as straight (1.2%)

### Relationship between IPV and depressive symptoms

The prevalence of depressive symptoms was 14.4% at month-12 (59/410) and month-24 (48/333). In the pooled analysis, depressive symptom prevalence was approximately three times higher in men who reported IPV victimization (lifetime or last year). There were similar, and stronger, associations for IPV perpetration measures (lifetime or last year) (Table 3). Compared to men who reported no experiences of IPV, the prevalence of depression was almost twice as high in men who reported unidirectional victimization and more than three times higher in men who reported both victimization and perpetration.

**Table 3:** Unadjusted and adjusted associations of IPV measures with depressive symptoms

*N* = 743 observations (using pooled 12/24 data in GEE models; *N* = 436 men)		Clinically significant depressive symptoms (PHQ-9 ≥ 10) 14.4% (107/743)		
		% (n/N)	Unadjusted PR [95% CI] *Overall p value*^b^	Adjusted^a^ PR [95% CI] *Overall p value*^b^
Lifetime IPV victimization	No/missing	8.9% (38/425)	1	1
	Yes	21.7% (69/318)	2.45 [1.63, 3.67]	2.57 [1.71, 3.86]
			***< 0.001***	***< 0.001***
IPV victimization in last year	No/missing	11.1% (70/630)	1	1
	Yes	32.7% (37/113)	2.82 [1.88, 4.22]	2.93 [1.96, 4.40]
			***< 0.001***	***< 0.001***
Lifetime IPV perpetration	No/missing	10.8% (65/603)	1	1
	Yes	30.0% (42/140)	2.83 [1.89, 4.22]	2.87 [1.91, 4.32]
			***< 0.001***	***< 0.001***
IPV perpetration in last year	No/missing	12.1% (83/688)	1	1
	Yes	43.6% (24/55)	3.40 [2.13, 5.41]	3.47 [2.13, 5.64]
			***< 0.001***	***< 0.001***
Combined lifetime IPV victimization/ perpetration^c^	Vict. & perp.	31.2% (39/125)	3.69 [2.33, 5.86]	3.87 [2.43, 6.16]
	Undirectional vict.	15.5% (30/193)	1.74 [1.07, 2.82]	1.83 [1.13, 2.98]
	Neither /missing	8.9% (38/425)	1	1
			***< 0.001***	***< 0.001***

^a^Age (included as four categories: < 25, 25–29, 30–39, 40+), born in the UK, sexual identity (gay or bisexual/straight), university education, and London clinic site

^b^*p* value by Wald test using GEEs. *p* values< 0.1 are indicated in bold

^c^Men who reported unidirectional IPV perpetration were excluded since the number of men reporting this measure (*n* = 11 at month-12 and *n* = 4 at month-24) was too small to allow for meaningful analysis, and these men did not fit into the ‘neither/missing’ category

### Relationship between IPV and sexual behaviour

**Fig. 3:**
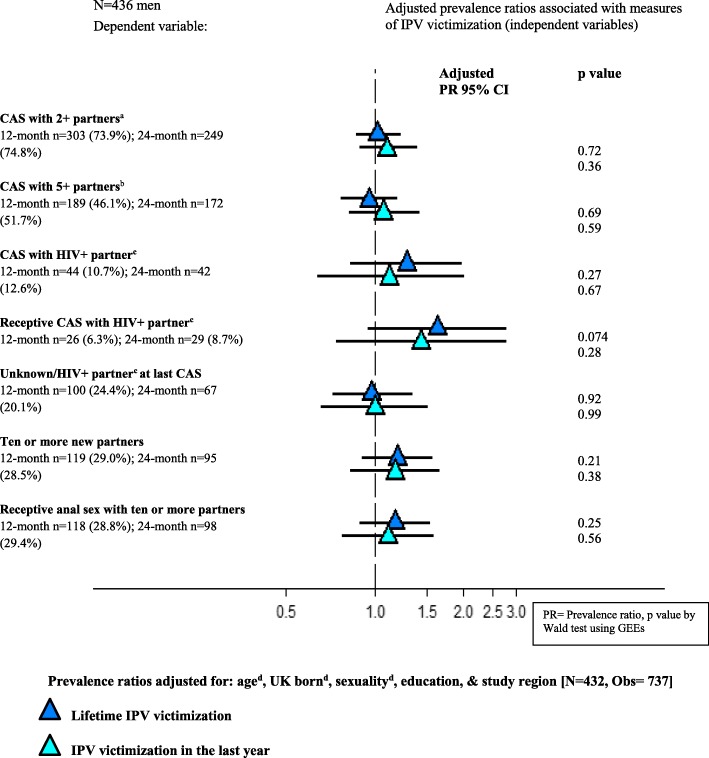
Adjusted associations of lifetime and past year measures of IPV victimization with sexual behaviours in the past three months among 436 men who participated in PROUD. ^a^ CAS with at least two receptive or at least two insertive CAS partners. ^b^ CAS with at least five receptive or at least five insertive CAS partners. ^c^ Not known to be on HIV treatment. ^d^ The model was fitted to include age in four categories (< 25; 25–29; 30–39; 40+), dichotomous UK born and self-reported sexual identity

**Fig. 4:**
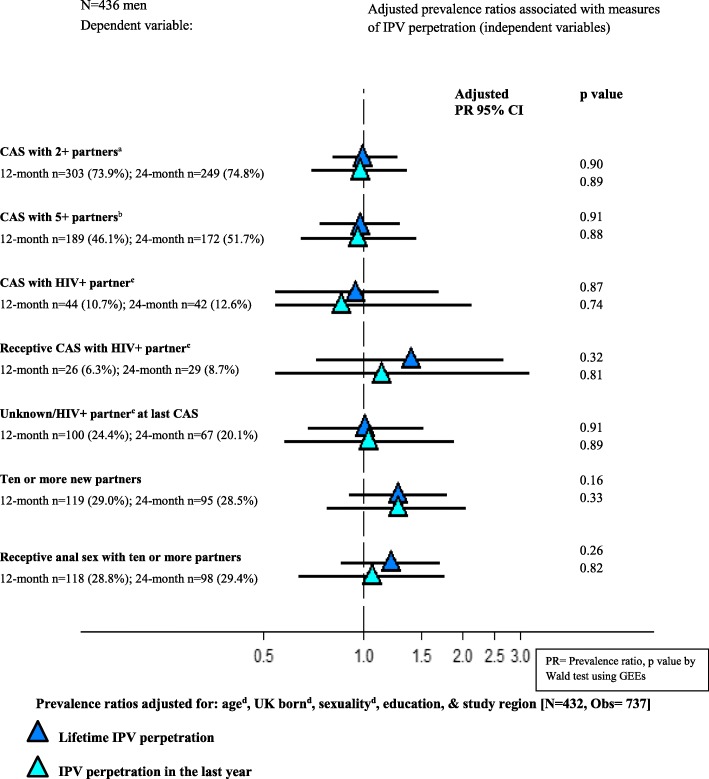
Adjusted associations of lifetime and past year measures of IPV perpetration with sexual behaviours in the past three months among 436 men who participated in PROUD. ^a^ CAS with at least two receptive or at least two insertive CAS partners. ^b^ CAS with at least five receptive or at least five insertive CAS partners. ^c^ Not known to be on HIV treatment. ^d^ The model was fitted to include age in four categories (< 25; 25–29; 30–39; 40+), dichotomous UK born and self-reported sexual identity

Measures of lifetime and past year IPV victimization and perpetration were not associated with sexual risk behaviours in GEE models (see Figs. 3 and 4). Unadjusted PRs were very similar to the adjusted PRs presented. There was some suggestion of a link between IPV victimization and receptive CAS with an HIV-positive partner not known to be on antiretroviral treatment, although this relationship did not reach statistical significance. Experiences of unidirectional IPV victimization and both victimization and perpetration were not associated with CAS measures or partner numbers in unadjusted or adjusted analyses.

### Impact of depression on relationship between IPV and sexual behaviour

Depressive symptoms were not associated with sexual risk behaviours in unadjusted or adjusted analysis (see Additional file 2). Associations between IPV measures and sexual behaviours remained the same after adjusting additionally for depressive symptoms. The relationship between lifetime IPV victimization and sexual behaviours was not different among men who reported depressive symptoms and men who did not; the interaction *p* values were not significant for any of the sexual behaviours (*p* ≥ 0.3).

### Handling missing data

A sensitivity analysis was undertaken excluding missing values when defining each variable. The findings were very similar to the main analysis.

## Discussion

This study found that measures of IPV were associated with younger age (perpetration only), sexualized drug use, internalized homophobia, group sex, and were strongly associated with depressive symptoms. IPV was not consistently associated with CAS measures among this study population.

The prevalence of IPV in the PROUD trial of GBMSM was very high: 44.9% (95% CI: 40.1, 49.7%) for lifetime IPV victimization and 15.6% (95% CI: 12.4, 19.5%) in the last year, and 19.5% (95% CI: 15.9, 23.7%) for lifetime IPV perpetration, and 7.8% (95% CI: 5.6, 10.8%) in the last year, at the 12-month questionnaire. There was some inconsistency of reporting of lifetime IPV between month-12 and 24 in PROUD. However, among those who reported IPV at month-12 but not 24, a common pattern of response was for men to report being frightened of the behaviour of a partner/having behaved in a manner that frightened a partner at month-12 and to respond ‘Never’ at month-24. Possibly, individuals may forget a single instance of IPV of this nature, view such an occurrence with less significance after a period of time, or ascribe a different meaning to it in the light of changes in the relationship or other circumstances.

The prevalence of IPV in PROUD is high when compared to the Crime Survey for England and Wales (2016) and the UK population-based Adult Psychiatric Morbidity Survey (2007, physical and/or emotional IPV only) whereby lifetime prevalence of IPV victimization was 10.1% (95% CI: 9.5, 10.7%) and 18.7% (95% CI: 17.1, 20.4%) for men respectively in the two studies, and 23.0% (95% CI: 22.2, 23.8%) and 27.8% (95% CI: 26.2, 29.4%) for women [9, 10]. However, both of these studies used a different assessment of IPV and did not present data separately for GBMSM. Estimates from PROUD are more in line with, although still higher than, those from a London GUM clinic cross-sectional study of gay- and bisexual-identified men (2010–2011, *N* = 519), which used the same measure of IPV (excluding ‘forced to have sex without a condom’): 33.9% (95% CI: 29.4, 37.9%) for lifetime IPV victimization and 16.3% (95% CI: 13.0, 19.8%) for lifetime IPV perpetration [7]. However, a qualitative study with 19 of these men, suggested that the survey results underestimated the prevalence of IPV [37]. PROUD estimates of lifetime IPV and IPV in the last year were also somewhat similar compared to those reported in two UK online samples of GBMSM that used different assessments of IPV. In one online study of 398 GBMSM [6], past year estimates of IPV were 8.5% (95% CI: 6.0, 11.7%) for physical IPV victimization, 3.3% (95% CI: 1.8, 5.5%) for physical IPV perpetration, 4.5% (95% CI: 2.7, 7.1%) for sexual IPV victimization, and 0.8% (95% CI: 0.2, 2.2%) for sexual IPV perpetration. In the other online study of 258 GBMSM [8], the prevalence of lifetime IPV victimization was 36.4% (95% CI: 30.6, 42.6%). Men may have been more likely to disclose IPV within the PROUD clinical trial setting, given more frequent contact with healthcare professionals and therefore opportunities for support and referral. Differences observed may also be attributed to the unique behavioural profile of the PROUD sample: there was a very high proportion who reported STIs and recreational drug use at baseline, very high levels of CAS at baseline and follow-up, and an exceptionally high incidence of HIV in the control group [15, 16], factors which may be associated with IPV. Therefore, IPV prevalence in PROUD may differ from other samples of GBMSM, and is not generalizable to the general GBMSM population in England.

In the current study, a trend was found with younger age and increasing prevalence of IPV perpetration, as has been found in other samples of GBMSM [38, 39]. Although no associations were found of sexual identity or ethnicity with lifetime and past year measures of IPV, the vast majority of the PROUD sample were gay identified and of white ethnicity. Furthermore, findings from a recent qualitative study suggest that dyadic inequalities including education and income differentials may serve as a means by which to establish power dynamics in same-sex male couples, and increase the risk of experiencing control and abuse from a partner [40]. The PROUD study sample likely lacked the statistical power to investigate these associations.

Having been a victim of IPV was very strongly associated with IPV perpetration in PROUD. It has been posited, across psychoanalytic theories, that exposure to/experiences of violence, abuse, and neglect, precede the perpetration of violence in future relationships [41–56]. The association between IPV victimization and IPV perpetration may be bidirectional. The very strong relationship observed in this study, may also reflect the phenomena of reciprocal IPV. In Johnson’s categorizations of IPV in opposite-sex partnerships, abuse may take one of four forms: (i) intimate terrorism, whereby one partner carries out abuse via a range of control tactics that are likely to escalate over time in a cyclical pattern of abuse, remorse, pursuit, and tension build-up, (ii) mutual violent control, whereby both partners are abusive and controlling, (iii) violent resistance, whereby one partner is violent and the other responds in violent self-defense, or (iv) situational couple violence, whereby one or both partners are abusive but the abuse is not attached to a pattern of escalating control [57]. In the current study, 16.8% of men reported lifetime experiences of IPV both as a victim and a perpetrator, although it was not possible to distinguish abuse carried out with the same or different partner. Further research is needed to examine whether the dynamics of IPV among same-sex male couples fit within Johnson’s four categories, and what processes are involved in the manifestation of these dynamics.

Evidence is accumulating which suggests that among UK (*N* = 398 [6]), U.S. (*N* = 1575 [4], *N* = 750 [5]), and Canadian (*N* = 186 [58]) samples of GBMSM, markers of internalized homophobia are strongly associated with measures IPV perpetration, including physical, [6, 58], emotional/psychological [58] and sexual [4, 5], in unadjusted analysis [4, 58], and after adjusting for socio-demographic and lifestyles factors [5, 6]. Associations have also been found with measures of IPV victimization in the U.S., including physical and sexual [5, 6]. In the current study, a marker of internalized homophobia was strongly associated with experiences of IPV victimization and IPV perpetration.

For sexual minority individuals, the internalization of anti-gay attitudes leads to feelings of worthlessness and negativity about the self, and may be linked to pervasive expectations of rejection, and non-disclosure of one’s sexual orientation. The link between internalized homophobia and IPV may be explained by exosystem factor theory and psychoanalytic theories. In exosystem factor theory, stress that is associated with exosystem level factors, the cultural or sub-cultural context in which development occurs, and is perceived to exceed one’s financial/emotional resources, is thought to be an important trigger for the perpetration of violence. This may occur in particular, against the backdrop of exposure to abuse/violence in childhood or early adolescence and lack of social support [59, 60]. Sexual identity is an exosystem level factor if an individual affiliates with a sexual minority population in a community. The stress associated with social pressure to conform to heteronormative behaviours may play some role in IPV perpetration among gay and plurisexual identified men. Enacting hegemonic masculinity via violent domination of one’s partner, may be used as a way of reconstructing a contested masculinity [61]. In psychoanalytic theories, abuse from significant individuals during formative years can manifest in persistent feelings of unworthiness, and an inability to regulate emotional responses and recognize/avoid abuse in adult intimate partnerships. Some individuals develop complex psychological defences necessary for survival, which become highly integrated into one’s personality structure [43, 46]. Although not possible in the current study, an understanding of the degree of exposure to abuse in childhood/adolescence, and emotional ties formed with primary caregivers, as well as levels of social support, may provide insight into why some men who experience internalized homophobia have violent partnerships while others do not.

In a UK study of GBMSM, IPV victimization in the last year was associated with past year use of ecstasy, LSD, cocaine, crack, heroin, or injected amphetamines (OR 1.7 95% CI: 1.16, 2.47, *p* = 0.006), after adjusting for socio-demographics [7]. Findings from PROUD suggest that sexualized drug use, which to a large extent may encompass the practice of chemsex, may be important in the context of IPV among GBMSM. Sexualized drug use (chemsex) may occur in group sexual settings. In this study, men who reported group sex were more likely to report IPV victimization in the last year, and there was some suggestion of a link with lifetime IPV perpetration. Group sex environments may leave some individuals vulnerable to mistreatment particularly if drugs are used, given their impact on inhibition and self-regulation [29, 62]. The relationship between recreational drug use and IPV may be bidirectional such that drugs are used as a form of self-medication and/or in order to induce a state of cognitive release [63].

Strong associations were found between IPV and depressive symptoms in the PROUD trial. This is in line with evidence from a recent meta-analysis of GBMSM [3], and suggests that experiences of IPV may have a lasting adverse impact on mental health. In PROUD, the association with depression was particularly strong for IPV perpetration, including men who both experienced and perpetrated abuse. Similarly, in the only UK study to have examined the link between IPV and depression among GBMSM, the prevalence of depression (HADS≥8) was significantly elevated among men reporting IPV perpetration in the past year versus those who hadn’t (20.7% vs. 11.5%), but not among men reporting victimization (past year or lifetime) [7]. In that study, after adjusting for socio-demographic factors, the association between IPV perpetration and depression was attenuated to borderline significance (OR 3.7 95% CI: 1.0, 14.6; *p = 0.060*). However, income was adjusted for, which may be highly correlated with both IPV and depression. When comparing these survey study findings to those provided during an interview, there was evidence to suggest that some men who abuse a partner do not report it on a survey questionnaire [37]. It is possible that for those men who do, the experience of IPV may have had a greater psychological impact. IPV perpetration is highly correlated with abusive experiences in childhood [43, 46, 59, 60, 64–66]. It is possible that a greater degree of exposure to violence during formative years may also explain a higher prevalence of depression among individuals who have carried out IPV. The relationship between depression and IPV may be bidirectional such that depressive symptoms heighten vulnerability to dysfunctional relationship dynamics, as adaptive coping mechanisms are distorted [67, 68].

Physical acts of violence directed towards an intimate partner do not often occur in isolation, frequently there is overlap with other forms of violence, including sexual abuse [69]. IPV may lead to a distortion of one’s perception of self-worth and ability to recognize dysfunctional relationship dynamics [56]. It is therefore plausible, that experiences of IPV with a previous partner may also lead to unwanted sex and CAS with other partners. In a recent meta-analysis [3], exposure to any kind of IPV was associated with CAS (pooled OR: 1.72 95% CI: 1.44, 2.05) and HIV seropositivity (pooled OR: 1.46 95% CI: 1.26, 1.69). In the current study, there was some suggestion of an association between lifetime IPV victimization and receptive CAS with an HIV-positive partner not known to be on antiretroviral treatment. However, measures of IPV were not found to be associated with any other measures of CAS or partner numbers in the PROUD trial. Similarly, no associations were found between depressive symptoms and sexual risk behaviour, despite the evidence for a relationship in other high-income country studies of GBMSM [70, 71]. There was no evidence from PROUD to suggest a synergistic effect of IPV and depression on sexual risk behaviour. The unique nature of the PROUD study population, GBMSM who reported very high levels of CAS, may explain why associations with CAS measures were not seen for depression or IPV. Perhaps IPV and depression do not explain why some men who engage in CAS have a higher number of CAS partners. It may be that other factors, with greater disinhibiting effects, such as higher levels of recreational drug use and/or personality traits associated with sexual compulsivity/sensation seeking, play a greater role in this context.

### The role of GUM services in addressing IPV

The UK National Institute for Health and Care Excellence recommends that trained staff in sexual health services ask about IPV as part of good clinical practice, even where there are no indicators of violence and abuse [72]. However, a recent UK survey (2010–2011) found that only 34.7% of 522 gay and bisexual GUM clinic attendees felt that ‘health professionals should ask all patients whether they have been hurt/frightened by a partner’, whereas 62.6% felt only some patients should be asked based on symptoms [37]. Further qualitative exploration revealed that men perceived the busy clinic environment as not conducive to asking all patients about IPV in a manner that would encourage disclosure. Conversely, some men felt that selective enquiry could be stigmatizing. At the very least sexual health services should display information on IPV, as well as train staff to recognize the common indicators, enquire sensitively about violence, and refer patients to further support within and outside of the health care setting. In the UK context this includes referral to domestic and sexual violence advisors (IDSVA) and local IPV services. General IPV support services for men include the ManKind Initiative and the Everyman Project, which offers counseling in London, as well as services specifically tailored to sexual and gender minorities, such as the Respect Phoneline and Galop LGBT Domestic Abuse Helpline, offering information and support. The IRIS ADViSE model, which encompasses training to enhance recognition, enquiry, and referral, has been shown to increase the IPV enquiry and identification rate among female GUM clinic attendees in a UK pilot study [73], and in a cluster randomized trial of women attending general practitioners in the UK, the identification of IPV and referral to specialist services [74]. In a recent RCT of Project WINGS, which aimed to provide effective IPV victimization screening, brief intervention, and referral to treatment services (SBIRT) for substance using women in New York, identification of IPV and receipt of IPV services was found to increase after 3-months of follow-up [75].

### Limitations

Information on the number and type of recreational drugs used in the past three months and higher risk alcohol consumption was only collected at the baseline questionnaire. Not being able to investigate other factors, which may be important in the context of IPV such as social support and financial security, was also a limiting factor. It was not possible to investigate data on dysfunctional relationships formed with primary caregivers in childhood/early adolescence. Not all PROUD participants were included in the IPV analysis due to missing questionnaires at months 12 and 24. Participants lost to follow-up may differ in terms of psychosocial factors. However, depression at baseline was not associated with loss-to-follow-up (overall or in each trial arm separately), and there was no difference between men with depressive symptoms and men without symptoms at the 12-month questionnaire in terms of completing the 24-month questionnaire (27.1% vs. 24.8% lost-to-follow-up respectively; *p*-value = 0.702). There was also no difference between men reporting experiences of lifetime IPV victimization and men who did not at the 12-month questionnaire in terms of completing the 24-month questionnaire (27.2% vs. 23.5% lost-to-follow-up respectively; p-value = 0.387). Even after including repeated observations in GEE models, given the relatively small sample size of the PROUD trial, the analysis may have lacked power to accurately detect the presence of some associations. GEEs were used for data-analysis in this paper, which treat the data as if it were cross-sectional, prohibiting inferences about causality. There is a need to conduct an adequately powered longitudinal study designed to address IPV among sexual minorities.

## Conclusions

In the PROUD trial of GBMSM at high-risk of HIV acquisition and seeking/taking PrEP, a very high lifetime prevalence of IPV was found. When addressing IPV, there is a need to also acknowledge and bring to the fore, the prevalence of violence in same-sex male couples. Training on IPV among same-sex couples should be enhanced in GUM settings with ongoing support and supervision for staff. Participation in the PROUD trial of PrEP efficacy did not appear to negatively influence experiences of IPV among GBMSM, and IPV prevalence was similar in both arms of the trial. IPV was strongly associated with sexualized drug use, internalized homophobia, and current symptoms of depression, but not with CAS measures. The impact of homophobia not only on one’s mental health but also on dynamics within intimate partnerships needs to be highlighted. Research is needed to better understand the effect sexual minority stress may have on IPV, including the magnitude of the effect and the direction and mechanisms of association.

## Additional files

Additional file 1: (PROUD annual questionnaire). (PDF 161 kb)

Additional file 2: (Table of associations between depressive symptoms and sexual behaviour measures). (PDF 86 kb)

## Abbreviations

BAME: Black, Asian, and minority ethnic
CAS: Condomless anal sex
CI: Confidence interval
CSA: Childhood sexual abuse
GBMSM: Gay, bisexual and other men who have sex with men
GEE: Generalized estimating equations
GHB/GBL: Gamma-hydroxybutrate/gamma-butyrolactone
GUM: Genitourinary medicine clinic
IDSVA: Domestic and sexual violence advisors
IPV: Intimate partner violence
IQR: Interquartile range
OR: Odds ratio
PHQ-9: Patient Health Questionnaire
PR: Prevalence ratio
PrEP: Pre-exposure prophylaxis
SBIRT: Screening, brief intervention, and referral to treatment services
