# Supplementary material for: Intimate partner violence, depression, and sexual behaviour among gay, bisexual and other men who have sex with men in the PROUD trial
Source: BMC Public Health. 2019 Apr 25;19:431. doi: 10.1186/s12889-019-6757-6 (PMC6482482; doi:10.1186/s12889-019-6757-6)
Supplement: Supplementary file 1 — (PROUD annual questionnaire). (PDF 161 kb) [file 12889_2019_6757_MOESM1_ESM.pdf]

Annual Sexual Behaviour Questionnaire

Trial no:	Initials:	Date of birth:	Date form completed:
P ☐☐☐☐☐☐	☐☐☐	☐☐/☐☐/☐☐	☐☐/☐☐/☐☐

Partnership Questions

1. How many current steady (sexual and emotional relationship) partners do you have? ☐☐

2. How many current casual (sexual relationship only) partners do you have? ☐☐

3. Among your current partners, what is the length of your longest relationship?

   ____ Years __ Months

Sexual Behaviour Questions

4. With how many different men have you had either been bottom (receptive, passive, he fucked you) or top (insertive, active, you fucked him) during anal sex in the last 90 days? ☐☐

5. With how many different men have you been bottom (passive) during anal sex in the last 90 days? ☐☐ *(If 0 please skip to question 9)*

6. Of the men in question 5, with how many were you bottom without using a condom? ☐☐ *(If 0 please skip to question 9)*

7. Of the men in question 6, how many did you know were HIV positive? ☐☐ *(If 0 please skip to question 9)*

8. Of the men in question 7, how many did you know were on HIV treatment? ☐☐

9. With how many different men have you been top (active) during anal sex in the last 90 days? ☐☐ *(If 0 please skip to question 13)*

10. Of the men in question 9, with how many were you top without using a condom? ☐☐ *(If 0 please skip to question 13)*

11. Of the men in question 10, how many did you know were HIV positive? ☐☐ *(If 0 please skip to question 13)*

12. Of the men in question 11, how many did you know were on HIV treatment? ☐☐

13. Of the men you've been either top or bottom with during anal sex in the last 90 days, how many were new partners? ☐☐ (This means men you had not had sex with before)

14. Think of the last time you had anal sex (top or bottom) with a man without a condom. These are reasons other men have given for not using condoms, please tick all that apply.

- ☐ I don't like using condoms
- ☐ He doesn't like using condoms
- ☐ Condoms weren't discussed
- ☐ We don't use condoms with each other but do with other partners
- ☐ Neither of us had any condoms
- ☐ I didn't consider myself at risk of HIV
- ☐ I was under the influence of alcohol
- ☐ I was under the influence of drugs
- ☐ I am faithful to him
- ☐ He is faithful to me
- ☐ It is more enjoyable without a condom
- ☐ I was only dipping
- ☐ I lose erections with condoms
- ☐ Other __________

15. Think of the last time you had anal sex (top or bottom) with a man without a condom. What was his HIV status?

- ☐ I don't know
- ☐ I thought he was HIV negative
- ☐ I thought he was HIV positive and on treatment
- ☐ I thought he was HIV positive and not on treatment
- ☐ I thought he was HIV positive and did not consider whether he was on treatment

Health and wellbeing Questions

16. Over the last 2 weeks, how often have you been bothered by any of the following problems?

	Not at all	Several days	More than half the days	Nearly every day
a) Little interest or pleasure in doing things	☐	☐	☐	☐
b) Feeling down, depressed or hopeless	☐	☐	☐	☐
c) Trouble falling or staying asleep, or sleeping too much	☐	☐	☐	☐
d) Feeling tired or having little energy	☐	☐	☐	☐
e) Poor appetite or overeating	☐	☐	☐	☐
f) Feeling bad about yourself – or that you are a failure or have let yourself or your family down	☐	☐	☐	☐
g) Trouble concentrating on things, such as reading the newspaper or watching television	☐	☐	☐	☐
h) Moving or speaking so slowly that other people could have noticed. Or the opposite being so fidgety or restless that you have been moving around a lot more than usual	☐	☐	☐	☐
i) Thoughts that you would be better off dead, or of hurting yourself in some way	☐	☐	☐	☐

Lifestyle Questions

17. We would like to know more about your social network. Please tick the box that applies to you:

	All or almost all	More than half	Less than half	Few	None
How many of your friends are gay or transgender/ have sex with men	☐	☐	☐	☐	☐
How many of your friends know that you are gay/transgender/have sex with men	☐	☐	☐	☐	☐
How many of your work colleagues know that you are gay/transgender/have sex with men	☐	☐	☐	☐	☐
How many of your close family members know that you are gay/transgender/have sex with men	☐	☐	☐	☐	☐

18. We would like to know more about where you meet <u>new</u> sexual partners. When did you last visit each of the following types of places to meet men (even if it is not typical for you):

	Within the last 4 weeks	Within the last 3 months	Within the last 6 months	More than 6 months ago	Never
Gay community centres, organisations, events or social groups	☐	☐	☐	☐	☐
Gay cafes, pubs and/or bars	☐	☐	☐	☐	☐
Gay nightclubs/discos	☐	☐	☐	☐	☐
Gay saunas	☐	☐	☐	☐	☐
Backrooms, sex clubs or gay party	☐	☐	☐	☐	☐
Bareback parties	☐	☐	☐	☐	☐
Cruising areas where men meet for sex with men	☐	☐	☐	☐	☐
Gay social networking websites (e.g. gay.com, gaydar.com, grindr, Manhunt, GayRomeo etc)	☐	☐	☐	☐	☐

19. All relationships go through good and bad times. We would like to know whether you have experienced any of the following behaviours in relationships with men. Please tick as many boxes per row that apply to you:

	Never	Yes – more than 1 year ago	Yes within the last year with former partner	Yes within the last year with current partner
Have you ever felt **frightened** of the behaviour of a partner	☐	☐	☐	☐
Have you ever needed to ask a partner's **permission** to work, go shopping, visit relatives, or visit friends (i.e. beyond the usual of being considerate to and checking with a partner)	☐	☐	☐	☐
Have you ever been **hit, slapped, kicked or otherwise physically hurt** by a partner	☐	☐	☐	☐
Have you ever been **forced to have sex** or made to engage in some sexual activity when you did not want to	☐	☐	☐	☐
Have you ever been **forced to have sex without a condom** when you did not want to	☐	☐	☐	☐
Have you ever behaved in a manner that has made a partner feel **frightened**	☐	☐	☐	☐
Has a partner ever needed to ask your **permission** to work, go shopping, visit relatives, or visit friends (i.e. beyond the usual of being considerate to and checking with a partner)	☐	☐	☐	☐
Have you ever **hit, slapped, kicked or otherwise physically hurt** a partner	☐	☐	☐	☐
Have you ever **forced a partner to have sex** or engage in some sexual activity when he did not want to	☐	☐	☐	☐
Have you ever **forced a partner to have sex without a condom** when he did not want to	☐	☐	☐	☐

a. **If you answered YES in the last year to any question above, do you think joining PROUD has influenced these behaviours?**

☐ No
☐ Yes in a positive way
☐ Yes in a negative way

20. How old were you the very first time you had any sexual contact with a male, or a male had any sexual contact with you? *Please provide an estimate if you cannot remember exactly* ☐☐

21. How old were you the very first time you had anal intercourse (top or bottom) with a male? *Please provide an estimate if you cannot remember exactly* ☐☐

22. We would like to know more about how you view your sexuality. Please tick the box that applies to you:

	Strongly agree	Agree	Neutral or uncertain	Disagree	Strongly disagree
Obviously effeminate homosexual men make me feel uncomfortable	☐	☐	☐	☐	☐
I feel comfortable in gay bars	☐	☐	☐	☐	☐
Social situations with transgender/gay men make me feel uncomfortable	☐	☐	☐	☐	☐
I feel comfortable being seen in public with an obviously transgender/gay person	☐	☐	☐	☐	☐
I feel comfortable discussing homosexuality in a public situation	☐	☐	☐	☐	☐
I feel comfortable being a transgender/gay man	☐	☐	☐	☐	☐
Homosexuality is morally acceptable to me	☐	☐	☐	☐	☐
Even if I could change my sexual orientation I wouldn't	☐	☐	☐	☐	☐

23. We would like to know more about the circumstances in which you have sex. When did you last do any of the following (even if it is not typical for you):

	Within the last 4 weeks	Within the last 3 months	Within the last 6 months	More than 6 months ago	Never
Had sex after having enough alcohol to feel drunk	☐	☐	☐	☐	☐
Had sex after using recreational drugs	☐	☐	☐	☐	☐
Had sex after using a substance that helps keep an erection	☐	☐	☐	☐	☐
Participated in group sex (sex with more than one other person on the same occasion)	☐	☐	☐	☐	☐
Paid a man for sex	☐	☐	☐	☐	☐
Received money for having sex	☐	☐	☐	☐	☐
Received drugs for having sex	☐	☐	☐	☐	☐
Received something else (such as a place to stay) for having sex	☐	☐	☐	☐	☐
Talked to a new sex partner about his HIV status before sex	☐	☐	☐	☐	☐
Talked to a sex partner about PrEP before sex	☐	☐	☐	☐	☐

24. We would like to know more about how you feel about your sex life as it is at the moment. Please tick the box that applies to you:

	Strongly agree	Agree	Neutral or uncertain	Disagree	Strongly disagree
I am happy with my sex life	☐	☐	☐	☐	☐
I am as sexually confident as I want to be	☐	☐	☐	☐	☐
I find it easy to say 'no' to sex I don't want	☐	☐	☐	☐	☐
The sex I have is always as safe as I want it to be	☐	☐	☐	☐	☐
I would like to have more sex	☐	☐	☐	☐	☐
I am usually sexually satisfied after sex	☐	☐	☐	☐	☐
I would like to have a, or more, steady partner(s)	☐	☐	☐	☐	☐
I would like to have a, or more, non-steady partners	☐	☐	☐	☐	☐
I would like more intimacy in my life	☐	☐	☐	☐	☐
I would like more love in my life	☐	☐	☐	☐	☐

25. We would like to know more about your use of PrEP <u>BEFORE</u> you joined the study. *Please tick one box per row to show how much each statement applies to you:*

	More than once	Once	Never
I bought Truvada privately (on the internet or elsewhere) and used it as PrEP	☐	☐	☐
I used someone else's Truvada tablets as PrEP	☐	☐	☐
I used someone else's antiretroviral tablets (not Truvada) as PrEP	☐	☐	☐

24. We are interested in your concerns about getting HIV whilst having sex. If you were to have sex with a man whose HIV status you did not know (unless otherwise specified), how worried would you later be about getting HIV in the following circumstances:

	Very Worried	Somewhat Worried	Not Worried
If you have unprotected receptive anal sex (no condom or PrEP)	☐	☐	☐
If you have unprotected insertive anal sex (no condom or PrEP)	☐	☐	☐
If you have unprotected receptive anal sex with a positive man who is on ART (no condom or PrEP)	☐	☐	☐
If you use post-exposure prophylaxis after unprotected receptive anal sex (no condom or PrEP)	☐	☐	☐
If you have condomless receptive anal sex while taking Truvada PrEP every day	☐	☐	☐
If you have condomless receptive anal sex while taking Truvada PrEP but had missed your previous days tablets	☐	☐	☐
If you have condomless receptive anal sex while taking Truvada PrEP but had missed several tablets that week	☐	☐	☐

Thank you for finishing the questionnaire.
Please place in the provided envelope and hand to a <u>doctor or nurse</u>
