# Supplementary material for: Intimate partner violence, depression, and sexual behaviour among gay, bisexual and other men who have sex with men in the PROUD trial
Source: BMC Public Health. 2019 Apr 25;19:431. doi: 10.1186/s12889-019-6757-6 (PMC6482482; doi:10.1186/s12889-019-6757-6)
Supplement: Supplementary file 2 — (Table of associations between depressive symptoms and sexual behaviour measures). (PDF 86 kb) [file 12889_2019_6757_MOESM2_ESM.pdf]

Additional file 2, Table 1: Unadjusted and adjusted associations of depressive symptoms with measure of CAS and partner numbers

Association of depressive symptoms (PHQ-9≥10) with sexual behaviour measures among 436 men (observations=743) using data at either the 12- or 24-month follow-ups in GEE models		Clinically significant depressive symptoms (PHQ-9≥10)
Dependent variables (past three months):		PR [95% CI] *Overall p value*[e]
CAS with 2+ partners[a]	Unadjusted	0.94 [0.74, 1.20] *p=0.622*
	Adjusted[d]	0.94 [0.74, 1.20] *p=0.632*
CAS with 5+ partners[b]	Unadjusted	0.81 [0.59, 1.11] *p=0.196*
	Adjusted[d]	0.80 [0.59, 1.10] *p=0.170*
CAS with HIV+ partner[c]	Unadjusted	0.86 [0.45, 1.62] *p=0.634*
	Adjusted[d]	0.87 [0.46, 1.64] *p=0.660*
Receptive CAS with HIV+ partner[c]	Unadjusted	0.75 [0.33, 1.73] *p=0.501*
	Adjusted[d]	0.75 [0.33, 1.73] *p=0.505*
Unknown/HIV+ partner[c] at last CAS	Unadjusted	1.15 [0.76, 1.73] *p=0.516*
	Adjusted[d]	1.17 [0.77, 1.77] *p=0.455*
Ten or more new partners	Unadjusted	0.96 [0.66, 1.40] *p=0.844*
	Adjusted[d]	0.96 [0.66, 1.41] *p=0.839*
Receptive anal sex with ten or more partners	Unadjusted	1.02 [0.71, 1.48] *p=0.913*
	Adjusted[d]	1.02 [0.70, 1.48] *p=0.925*

[a] CAS with at least two receptive and/or insertive CAS partners.
[b] CAS with at least five receptive and/or insertive CAS partners.
[c] Not known to be on HIV treatment.
[d] Age (included as four categories: <25, 25-29, 30-39, 40+), born in the UK, sexual identity (gay or bisexual/straight), university education, and clinic site.
[e] p value by Wald test using GEEs
